# Effect of Different Dietary Doses of Black Soldier Fly Meal on Performance and Egg Quality in Free-Range Reared Laying Hens

**DOI:** 10.3390/ani14223340

**Published:** 2024-11-20

**Authors:** Carlos Romero, Juan Carlos Cenalmor, Susana Chamorro, César Redondo

**Affiliations:** 1 Facultad de Ciencias y Artes, Universidad Católica Santa Teresa de Jesús de Ávila (UCAV), Calle Canteros s/n, 05005 Ávila, Spain; 2 Granja Monte Encinar, El Barraco, 05110 Ávila, Spain; 3 Unidad de Fisiología Animal, Departamento de Genética, Fisiología y Microbiología, Facultad de Ciencias Biológicas, Universidad Complutense de Madrid, Calle José Antonio Novais 12, 28040 Madrid, Spain; schamorr@ucm.es

**Keywords:** *Hermetia illucens*, hen, laying performance, egg quality, fatty acid profile, vitamin

## Abstract

(1) Background: Given the problems currently posed by the use of soybean meal in poultry feeding, its replacement with black soldier fly (BSF) meal may be a suitable strategy. Therefore, this study evaluated the effect of this dietary replacement on laying performance, egg quality, and yolk nutritional composition in free-range reared hens. (2) Methods: Three diets were formulated: a control diet with 210 g/kg of soybean meal, a diet with 105 g/kg of soybean meal and 80 g/kg of BSF meal, and a diet with 160 g/kg of BSF meal. This eight-week-long study was performed with 126 Bovans Brown hens aged 36 weeks (six replicates of seven hens each per dietary treatment); (3) Results: The laying performance of hens was not influenced by the diet consumed. Neither was the yolk content in fat, protein, cholesterol, choline, B vitamins, and cholecalciferol affected. The dietary inclusion of BSF meal made yolk colour score and albumen Haugh units decrease (*p* < 0.001) by 51.1% and 12.0%, respectively. It also reduced the yolk content in retinol (by 10.1%, *p* = 0.0037), but it increased that of α- and γ-tocopherol (by 27.6%, *p* = 0.014, and 25.8%, *p* = 0.0077, respectively). A negative linear effect (*p* < 0.001) was observed on yolk zinc concentration and on the proportions of polyunsaturated and ω-3 fatty acids with increasing dietary dose of BSF meal; (4) Conclusions: Soybean meal could be fully replaced by BSF meal in the diet of hens without affecting the laying rate or the egg weight. However, the dietary inclusion of BSF meal led to paler yolks and impaired albumen quality, while it decreased the content of retinol and zinc and the proportions of polyunsaturated and ω-3 fatty acids in egg yolk.

## Simple Summary

In recent years, the European Commission has approved the use of farmed insects in poultry feeding. Among the different farmed insects allowed, the black soldier fly (*Hermetia illucens*) stands out for being a dense source of nutrients and for its ability to transform most organic wastes, such as household food leftovers and livestock manure, into a rich source of essential amino acids, enabling thus the recycling of large amounts of residues. Currently, the main protein source used in the feed of laying hens is soybean meal. However, the use of soybean meal entails several concerns like deforestation, high carbon footprint, and its origin from genetically modified seeds. In the present research work, it was proven that soybean meal can be fully replaced by black soldier fly meal in the diet of hens without affecting the laying performance or the egg weight. This replacement also led to higher yolk concentration in vitamin E. Nonetheless, the dietary inclusion of black soldier fly meal also implied some impairment in egg quality, such as lower yolk colour score, worse albumen quality, reduced yolk concentrations in zinc and vitamin A, and decreased percentages of polyunsaturated and ω-3 fatty acids in egg yolk.

## 1. Introduction

Because of their high content in essential amino acids, their richness in vitamins A, D, B_7_, B_9,_ and B_12,_ and their high concentration in minerals like phosphorus, iron, and zinc, hen eggs constitute an affordable high-quality source of essential nutrients for human beings [1,2,3]. Eggs also represent an important font of a wide array of high biological value proteins, with more than 100 different types of proteins having been identified in egg yolk [4]. Furthermore, the consumption of egg yolks enables an adequate intake of essential fatty acids since linoleic acid has repeatedly been reported to be one of the most abundant fatty acids in egg yolk [5,6]. Moreover, when feeding laying hens with a diet including ingredients rich in ω-3 fatty acids such as linseed, microalgae or fish oil, the fatty acid profile of egg yolk becomes even healthier given that the proportion of essential ω-3 fatty acids is increased, and ω-6/ω-3 ratios between 2 and 3 are achieved [7,8]. Indeed, hen eggs can help meet the nutritional requirements of an increasing world population that is expected to reach 9 billion people by 2050 [9]. Especially, the consumption of eggs can be of utmost importance for the fulfilment of the vitamin requirements of children and pregnant women [10]. Accordingly, the global number of laying hens and the number of eggs in shell produced worldwide increased, respectively, by 63.0% and 70.4% from 2000 to 2020 [11].

Currently, the main source of protein used in the feed of laying hens is soybean meal. The increase in the number of laying hens in the world is hence leading to a higher demand for soybeans. Most soybean meal consumed in poultry feeding in Europe is imported from South American countries, such as Brazil and Argentina. The cultivation of soybeans in these countries entails nowadays several societal and environmental concerns like deforestation of native forests, long-distance transportation, high carbon footprint, and even human rights violations [12,13].

Furthermore, in recent years, a clear shift has occurred in the European Union as regards the rearing system of laying hens. While the number of laying hens housed in enriched cages dropped by 30.7% from 2011 to 2023, the number of laying hens reared in free-range and organic conditions rose, respectively, in the same period by 142 and 123% [14]. For the production of organic eggs, higher dietary doses of soybean meal are needed in order to meet the requirements of birds in essential amino acids, as compared with the diets of hens housed in cages, since in organic conditions dietary supplementation with synthetic amino acids is not allowed. Nonetheless, the use of American soybean meal poses a problem in organic poultry farms because most imported soybean products originate from genetically modified seeds. Consequently, the provision of soybean meal for feed mills manufacturing compound feeds for organically-reared laying hens is not always easily achieved.

For all the reasons mentioned, it seems pertinent to look for an alternative protein source that could replace soybean meal in the feed of laying hens. The use of farmed insects in poultry feed has been recently authorised by the European Commission [15]. Indeed, poultry are insectivorous animals, and accordingly, it has been reported that the red jungle fowl, the wild ancestor of domestic chickens, feeds on a wide variety of insects in nature [16]. Among the different farmed insects permitted in the European Union for the feeding of poultry, the black soldier fly (BSF, *Hermetia illucens*) stands out for being a dense source of crude protein, metabolisable energy, calcium and phosphorus [17,18,19,20] and for its ability to transform most organic wastes such as household food leftovers and livestock manure into a rich source of essential amino acids [21,22], enabling thus the recycling of large amounts of residues and contributing thereby to the achievement of Target 12.5 of the 2030 Agenda’s Sustainable Development Goals [23]. Moreover, the rearing of BSF can be deemed more sustainable and eco-friendly than the raising of soybeans because insect farming is land-sparing. Finally, another advantage of using insects, instead of soybean, as main protein source in the diets of poultry is that insects are not part of the diet of human beings in Western societies, owing to cultural and ethnic perceptions, and hence, unlike what happens with soybean, the use of BSF in poultry feeding does not imply a competition with human food.

The partial or even the full replacement of soybean meal with BSF meal in the diet of laying hens has already been tested, but most studies available have been conducted with laying hens housed in cages [24,25,26,27] or in indoor aviaries [28] and only very few ones have been carried out with laying hens kept in free-range conditions [29]. However, given the current tendency in European countries towards increasing production of free-range eggs, it seems pertinent to broaden the knowledge about the effects of the dietary inclusion of BSF meal on productive performance and egg quality in laying hens reared in free-range conditions.

Therefore, the present research work aimed at assessing the effect of the inclusion at three different doses (0, 80 and 160 g/kg) of a partially defatted BSF meal in the diet of free-range kept laying hens on productive results, egg quality indicators (thickness and proportion of eggshell, yolk colour score, albumen Haugh units), and yolk nutritional composition (fat, crude protein, cholesterol, choline, vitamins, minerals and carotenoid pigments).

## 2. Materials and Methods

### 2.1. Experimental Design

The trial of the current research work was performed in a private commercial farm rearing laying hens in free-range conditions in El Barraco (Ávila, Spain). The trial lasted eight weeks, starting on 15 July 2024. One hundred and twenty-six Bovans Brown hens, aged 36 weeks at the beginning of the trial (average live weight: 1843 ± 24.1 g), were used for this study. Birds were at all times handled in accordance with the guidelines for the Care and Use of Animals for Scientific Purposes of the Ministry of Agriculture, Fishery and Food of Spain. Furthermore, it should be pointed out that no samples were taken on the hens and that no bird was killed during the trial. This experiment only implied egg collection in order to assess the quality parameters and nutritional composition of the eggs. This research work was granted ethical approval by the Ethics Committee of the Regional Government of Castilla y León (Spain).

In this study, three different dietary doses (0, 80, and 160 g/kg) of a partially defatted BSF meal were tested. The BSF meal was purchased at Insectius (San Andrés de Llavaneras, Barcelona, Spain). The BSF meal was included in the diet of laying hens to partially or completely replace soybean meal as the main protein source of the feed. Table 1 shows the nutritional composition of the soybean meal and the BSF meal included in the diets of this experiment. Forty-two hens were randomly allocated to each dietary treatment. Prior to the beginning of the trial, hens were given a two-week adaptation period to the experimental diets. Table 2 provides the ingredient and nutrient compositions of these diets that were formulated to be isocaloric and isonitrogenous and to contain the same amount of calcium, phosphorus, and sodium. Diets were devoid of synthetic pigments. Hens were offered ad libitum access to feed (provided in mash form) and water throughout the whole trial. The forty-two laying hens of each treatment were randomly distributed to six outdoor fenced parks of 60 m^2^ equipped with a shed of 12 m^2^ (each park constituted a replicate; six replicates per dietary treatment; seven hens per replicate). Hens were allowed 24-h free access to the shed (sheds were kept open for the entire day). Feeders, drinking troughs, and nests were placed within the sheds.

### 2.2. Hen Performance Recording

On a daily basis, all eggs laid were collected manually, counted, and weighed. Subsequently, daily egg production (%) was determined per replicate by dividing the total number of eggs collected on a day by the number of hens in the replicate and then by multiplying by 100. Feed consumption by hens from each replicate was monitored weekly and divided by forty-nine to get the daily feed intake per hen. Next, daily egg mass was calculated by multiplying the daily egg production (%) by the average egg weight divided by 100. Thereafter, the feed conversion ratio per replicate was determined by dividing feed intake by egg mass.

### 2.3. Egg Collection and Egg Quality Assessment

On the first three days of week eight of the trial, eighteen freshly laid eggs were collected per replicate (108 eggs collected per dietary treatment). In six out of the eighteen eggs originating from each experimental replicate (36 eggs per dietary treatment), the following variables were measured: egg weight, thickness and proportion of eggshell, yolk colour score, and albumen Haugh units. Yolk colour was determined with the Roche Yolk Colour Fan, and results were expressed according to the standard DSM Roche Fan values (from 1 for light yellow to 15 for orange). The albumen height in the eggs was determined using a QCH device (TSS, York, UK). Then, Haugh units were calculated with the following formula: Haugh units = 100 × log (h − 1.7 × w^0.37^ + 7.57), where h = albumen height (mm) and w = egg weight (g) [30].

The weight of the shell (including testaceous membranes) was measured after rinsing and drying the shells at room temperature for 24 h. The eggshell proportion was calculated by dividing the weight of the dried shell by the total egg weight. Shell thickness was measured at the equator of eggs with a digital Mitutoyo micrometre (Kawasaki, Japan).

Yolks from four eggs of the same dietary treatment were collected and pooled (nine pools per dietary treatment). The yolk pools were frozen at −80 °C, lyophilised using a Daihan Scientific Unifreez FD-8 freeze-dryer (Gangwon-do, South Korea) and later on, used for the quantification of yolk content in fat, crude protein, cholesterol, choline, biotin, folate, cobalamin, retinol, cholecalciferol, α-and γ-tocopherol, phosphorus, iron, zinc and carotenoid pigments and for the assessment of yolk fatty acid profile.

With the aim of evaluating the effect of egg storage on albumen quality in the different experimental groups of birds, the remaining 72 eggs per dietary treatment (twelve eggs per replicate) were stored in darkness at a constant temperature of 4 °C. At the close of 14 days of storage, 36 eggs (six eggs per replicate) were taken and broken for measurement of albumen Haugh units. The same protocol was applied to the remaining 36 eggs at the end of 30 days of storage.

### 2.4. Chemical Analyses

Chemical analyses of samples were performed in triplicate. Dry matter (930.15), crude protein (976.05), crude fibre (978.10), starch (996.11), ash (942.05), cholesterol (994.10), carotenoids (941.15), calcium, phosphorus and sodium (985.01), cholecalciferol (2002.05) and biotin (2016.02) were conducted in keeping with the methods of the Association of Official Analytical Chemists [31]. Fat was analysed by Soxhlet analysis (method 4.B) after 3 M HCl acid hydrolysis [32]. The characterisation of the fatty acid profile was completed following method 996.06 of AOAC [31] and as reported by Romero et al. [33]. Fatty acids present in the samples were methyl esterified, and thereafter, the fatty acid methyl esters were analysed with a gas chromatograph (Agilent 7820A) fitted with a flame-ionisation detector and an Agilent HP-88 column (60 m × 250 μm × 0.2 μm). The fatty acid analyses were conducted in duplicate. Amino acids were analysed by acid hydrolysis (for methionine and cysteine, samples were previously oxidised with performic acid; for tryptophan, samples were hydrolysed with barium hydroxide and water) followed by HPLC (AOAC method 982.30). Choline content was analysed using liquid chromatography coupled with tandem mass spectrometry, as described by Hirakawa et al. [34]. Analyses of cobalamin were completed following the protocol of Tekin et al. [35]. Folate concentration was determined via HPLC in accordance with the method described by Hebert et al. [36]. Quantifications of retinol, α-tocopherol, and γ-tocopherol contents were performed as explained in a previously published study by our team [37]. Finally, the concentration of iron and zinc was determined by inductively coupled plasma mass spectrometry.

### 2.5. Statistical Analysis

Data of variables measured were subjected to an analysis of variance (ANOVA) by using the general linear model procedure of SAS (Version 9.4, SAS Institute Inc., Cary, NC, USA) and with the diet consumed by hens as the main source of variation. When the dietary effect was declared significant (*p* < 0.05), comparisons among the treatment means were made using a t-test. Linear and quadratic effects of the dietary dose of BSF meal were also analysed. Non-orthogonal contrasts were used to evaluate the difference between the value obtained with the control diet and the combined value of the diets including BSF meal. Furthermore, results on albumen Haugh units were also analysed using the general linear model procedure of SAS with the diet, the egg storage time and their interaction as main sources of variation.

The batch of seven laying hens constituted the experimental unit for egg-laying performance results (variables measured: egg laying rate, egg weight, egg mass, feed intake and feed conversion ratio). The egg represented the experimental unit for the following egg quality parameters: thickness and proportion of eggshell, yolk colour score and albumen Haugh units. Finally, a pool of four yolks originating from eggs of the same dietary treatment corresponded to the experimental unit for the yolk contents in fat, crude protein, cholesterol, choline, vitamins, minerals and carotenoid pigments and for the fatty acid profile.

## 3. Results

### 3.1. Laying Hen Performance

Table 3 shows the effect of the dietary inclusion of BSF meal on the productive performance of laying hens. None of the parameters measured was affected by the diet consumed by hens. Daily egg production, average egg weight and feed conversion ratio averaged, respectively, 93.4%, 61.8 g and 2.20. No hen died during the trial in any of the replicates.

### 3.2. Egg Quality

**Figure 1:**
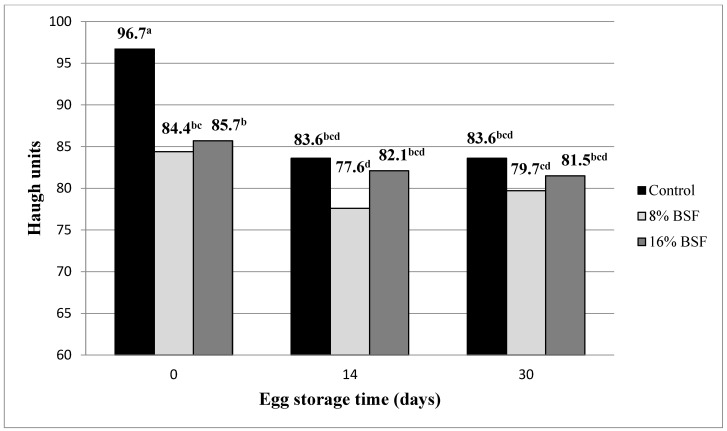
Effect of egg storage time on albumen Haugh units depending on the diet consumed (SEM = 1.27; n = 108 eggs at each day; *p*_time_ < 0.001; *p*_diet_ < 0.001; *p* _time x diet_ = 0.042). Diets: Control diet; 8% BSF meal: diet including 80 g/kg of defatted black soldier fly meal; 16% BSF meal: diet including 160 g/kg of defatted black soldier fly meal. Eggs were stored in darkness at a constant temperature of 4 °C. ^a–d^ Means with different superscripts differ significantly (*p* < 0.05).

Results on the egg quality parameters are provided in Table 4. Again, it was found that egg weight was not influenced by the inclusion of BSF meal in the diet of hens (61.7 g, on average). Also, shell thickness (358 μm, on average) and shell proportion (11.6%, on average) remained unaffected by the diet consumed by laying hens. Nevertheless, the inclusion of BSF meal in the diet made yolk colour score and albumen Haugh units of freshly laid eggs decrease (*p* < 0.001), respectively, by 51.1% and 12.0%, with no significant difference being detected for these traits between the two dietary doses of BSF meal. The interaction between the dietary treatment and the egg storage time was revealed to be significant (*p* = 0.042; Figure 1) since, contrary to what happened in freshly laid eggs, in eggs that had been stored for 14 and 30 days no significant differences were found among diets for the albumen Haugh units (on average, 81.1 and 81.6 Haugh units at 14 and 30 days of storage, respectively).

### 3.3. Egg Nutritional Composition

The effect of the dietary dose of BSF meal on fat, crude protein, cholesterol, choline, vitamins, carotenoids and minerals contents in the egg yolk is reported in Table 5. The egg yolk content in fat (62.9% DM, on average), crude protein (31.9% DM, on average), cholesterol (1988 mg/100 g DM, on average) and choline (592 mg/100 g DM, on average) were not influenced by the inclusion of BSF meal in the diet of laying hens.

The yolk content in none of B vitamins determined in the current research work was either affected by the diet fed to the hens, with the average concentrations of biotin, folate and cobalamin being, respectively, 1.39 μg/g DM, 28.3 μg/100 g DM and 5.60 μg/100 g DM.

As regards fat-soluble vitamins, the presence of BSF meal in the diet reduced, irrespective of the dietary dose used, the egg yolk content in retinol (9.71 vs. 10.8 μg/g DM, *p* = 0.0037), whereas it increased that of γ-tocopherol (9.75 vs. 7.75 μg/g DM, *p* = 0.0077). The egg yolk concentration in α-tocopherol was only increased with the highest dietary dose of BSF meal, as compared with the control group (148 vs. 116 μg/g DM, *p* = 0.0136).

A positive linear effect was observed on the egg yolk content in total carotenoids (up to 60.6%, *p* = 0.0031), lutein (up to 23.0%, *p* = 0.0091) and zeaxanthin (up to 77.5%, *p* = 0.0010) with increasing dietary dose of BSF meal. The egg yolk concentration of α-,β-cryptoxanthin rose up to an average value of 1.61 mg/kg in eggs from hens fed the diets containing BSF meal, whereas α-,β-cryptoxanthin was not detected in eggs from hens fed the control diet.

Concerning minerals content in the egg yolk, neither iron concentration (111 mg/kg DM, on average) nor that of phosphorus (1.09% DM, on average) was affected by the dietary treatment, but zinc content in the egg yolk linearly decreased (from 79.0 down to 70.7 mg/kg DM, *p* < 0.001) with increasing dietary dose of BSF meal.

The fatty acid profile of egg yolks is provided in Table 6. The proportion of saturated fatty acids in egg yolks did not differ significantly among experimental diets (32.7%, on average). However, the proportion of monounsaturated fatty acids rose (from 41.3% up to 48.9%, *p* < 0.001), while that of polyunsaturated fatty acids decreased (from 25.9% down to 18.1%, *p* < 0.001), as the dietary dose of BSF meal increased. Indeed, both the proportion of ω-6 fatty acids and that of ω-3 fatty acids decreased (*p* < 0.001) in egg yolks as the dietary content in BSF meal became greater. The effects observed were mainly due to a quadratic increase in the proportion of oleic acid (from 37.8% up to 43.7%, *p* < 0.001) and a quadratic reduction in the proportion of both linoleic acid (from 21.9% down to 15.0%, *p* = 0.011) and eicosatrienoic acid (from 2.17% down to 1.61%, *p* = 0.019) with increasing dietary dose of BSF meal.

## 4. Discussion

### 4.1. Nutrient Composition of the Black Soldier Fly Meal

As compared with the soybean meal, the partially defatted BSF meal tested in the current research work stood out for its higher content of crude protein, lysine, calcium and phosphorus. Despite varying contents in fat in the different samples of BSF meal evaluated in other research works, the results obtained in the current study for the contents of crude protein and lysine in BSF meal are consistent with those reported in previously published papers [19,20,24,38]. The calcium and phosphorus contents in BSF meal appear to be more variable among studies, but still, the amounts found in the present research are in keeping with those obtained by other researchers [19,39]. The fibre content was also higher in BSF meal than in soybean meal (103 vs. 39 g/kg). This difference can be mainly ascribable to the high chitin content (around 70 g/kg) in BSF meal [38,40]. Chitin is a polysaccharide present in the exoskeleton of arthropods.

As regards the fatty acid profile, the BSF meal showed a proportion of saturated fatty acids higher than that of soybean meal, whereas the proportion of polyunsaturated fatty acids was lower in the BSF meal. These differences in the fat profile between BSF and soybean meals are mainly due to the higher percentage of lauric acid in BSF meal (30.9% in BSF meal, while not detected in soybean meal) and a much lower percentage of linoleic acid in BSF meal than in soybean meal (13.7 vs. 55.2%). The fatty acids detected in higher proportion in the BSF meal used in this trial were lauric acid, palmitic acid and oleic acid. Even if it has been reported that the fat profile in BSF depends on the substrate used to raise BSF [41], the latter fatty acids (C12:0, C16:0 and C18:1 ω-9) have consistently been found to be the predominant fatty acids in BSF in different studies [42,43,44]. Indeed, a fatty acid profile very similar to that found in this work was reported for BSF meal by Secci et al. [25].

### 4.2. Hen Performance

Results found in the current study revealed that the complete replacement of soybean meal with BSF meal in the diet of laying hens could be done without affecting the egg-laying performance of birds. Important parameters such as the daily egg production, the average egg weight and the feed conversion ratio remained unaffected by the change in the main dietary protein source. This finding is consistent with previous scientific works [26,40,45] in which it was also observed that hen egg production or feed conversion ratio was not affected when soybean meal was completely replaced in the diet of laying hens by a partially defatted BSF meal with a nutrient composition similar to that of the BSF meal used in the current study. Concerning egg weight, different effects have been reported. While Heuel et al. [40] reported a lack of effect of complete dietary replacement of soybean meal with BSF meal on egg weight, Mwaniki et al. [26] found that average egg weight was reduced by 2.25% with this replacement of the main dietary protein source. In the latter study, the diet in which soybean meal had been completely replaced with BSF meal contained 35% less linoleic acid. This could have accounted for the reduction in egg weight, as it is well known that the dietary content in linoleic acid influences egg weight [46,47,48]. In the present research work, the diet including BSF meal at 160 g/kg contained 41.4% less linoleic acid than the control diet, but still, the content of linoleic acid (2.50%) in the 16% BSF diet remained well above the recommended levels of linoleic acid (1.35–2.0%) to maximise egg weight [49,50], whereas in the study of Mwaniki et al. [26] the dietary content in linoleic acid dropped down to a rather low value (1.27%) that may have been insufficient to ensure optimal egg weight. Hence, this could explain why, in the present trial, egg weight was not significantly reduced. Furthermore, it should be noted that even if the dietary inclusion of BSF meal leads to higher dietary contents of sulphur amino acids, the actual dietary available content of these amino acids may not be that high since the chitin present in BSF meal, indigestible for monogastric animals, may hinder the digestion of proteins [51] and thereby reduce the dietary amount of digestible amino acids [21]. This could affect the availability of dietary methionine and cysteine and negatively influence egg weight. Therefore, attention should be paid to the dietary contents in digestible amino acids, especially as regards indispensable amino acids, when soybean meal is replaced with BSF meal.

Lastly, irrespective of the diet consumed, productive results achieved by the hens of the present trial were in keeping with the targeted performance provided in the product guide of free-range reared Bovans Brown hens [52]. Besides, taking into account the absence of mortality and digestive troubles such as diarrhoea, it could be surmised that eating BSF meal did not cause any adverse effect on the laying hen’s health status.

### 4.3. Egg Quality

As previously detected by other researchers [26,40,45,53,54], the dietary inclusion of BSF meal had no influence on eggshell thickness in the present study. All diets of this research work met the recommendation of dietary calcium levels for high-yielding laying hens [55], and the ratio calcium:phosphorus was maintained more or less constant among diets. In the current work, it was observed that irrespective of the dietary dose used, the inclusion of BSF meal in the diet of laying hens negatively affected both yolk colour score and albumen Haugh units in freshly laid eggs. It may seem paradoxical that the consumption of diets including BSF meal resulted in a decrease in yolk colour score when these same diets also led to higher concentrations of carotenoids in the yolk. Even if carotenoids are responsible for egg yolk colour, it is not the amount of carotenoids in the yolk that determines its colour but rather the kind and proportions of carotenoids [56,57]. Indeed, paler egg yolks have been found to contain more carotenoids than yolks with higher scores on the DSM Yolk Colour Fan [58,59,60]. Likewise, in the present research work, feeding diets including BSF meal resulted in higher yolk concentrations of xanthophylls lutein and zeaxanthin, but this was not associated with a more orange hue. Perhaps the latter was due to a lower content of red carotenoids (e.g., canthaxanthin) in the diets containing BSF meal. Actually, Lokaewmanee et al. [61] detected in parallel a decrease in the redness of yolks and lower yolk colour score when the diet fed to the hens contained BSF larvae, and this happened regardless of the dietary dose (either at 10, 20 or 30 g/kg) at which BSF larvae were included. Given that consumers attach great importance to yolk colour [62], especially in Mediterranean countries where people prefer high scores (13–14), future research should focus on characterising and quantifying red carotenoids in BSF meal and in the yolk of eggs laid by hens fed diets including this insect meal. Some strategies should be implemented to avoid the decrease in yolk colour score when soybean meal is replaced with BSF meal in the diet of laying hens.

As aforementioned, the dietary inclusion of BSF meal also caused an impairment of albumen quality in the current study, with no difference between dietary doses of BSF meal. This result is surprising and inconsistent with previous studies tackling the dietary replacement of soybean meal or fish meal with BSF meal since, in general, all researchers have reported a lack of effect of dietary inclusion of BSF meal on albumen Haugh units [26,40,45,53,54]. The main factor influencing albumen Haugh units is hen age, and little effect is attributed to diet nutritional composition [63]. In the current study, birds had exactly the same age in all replicates, so age could not account for the difference detected. Authors have decided to conduct new trials in the future to evaluate again the effect of dietary inclusion of BSF on albumen Haugh units because, currently, they are unable to find an explanation for the difference detected. Nevertheless, it should be pointed out that this difference was only found to be significant in freshly laid eggs, and then this significance was no longer detected in stored eggs.

### 4.4. Egg Nutritional Composition

Apart from water, the main constituents of egg yolk are fat and protein [2]. The yolk contents in fat and protein obtained in the eggs of this work did not differ significantly among dietary treatments and fell within the range of usual values of yolk concentration in these nutrients [7]. Neither did fat and protein concentrations in the yolk differ in previously published studies because of partial [54] or complete [25] dietary replacement of soybean meal with BSF meal.

The inclusion of BSF larvae in the diet of laying hens [29] or that of BSF larva fat in the diet of broiler chickens [64] had no influence on the blood cholesterol concentration of these birds. Furthermore, feeding hens with diets including full-fat BSF meal did not result in any significant change as regards yolk content in cholesterol in the study of Thao et al. [65]. Accordingly, neither was the yolk content in cholesterol affected by the dietary inclusion of BSF meal in the present work. Indeed, Naber [66] stated that changes in diet composition result in little impact on egg cholesterol content. In this sense, the results obtained in this work for the yolk cholesterol concentration are similar to those obtained in other studies in which very different diets were fed to the laying hens [7,67,68,69].

To the authors’ knowledge, no research work has hitherto reported the effect of the dietary inclusion of BSF products on yolk content in choline or in B vitamins. Therefore, no comparison was possible with other trials. In the present study, yolk content in choline, biotin, folate and cobalamin remained unaffected by the inclusion of BSF meal in the diet of laying hens, and the values obtained for these parameters fell within the range of usual yolk concentrations in choline [34,68], biotin [70], folate [36,71] and cobalamin [7,72].

Concerning fat-soluble vitamins, the inclusion of BSF meal in the diet reduced yolk concentration in retinol, increased that of α- and γ-tocopherol and had no effect on the content in cholecalciferol. Also, for these vitamins, there is a dearth of studies assessing the effect of the dietary inclusion of BSF products on their yolk content. In agreement with what was found in the present research work, Secci et al. [25] reported an increase in yolk γ-tocopherol content when soybean meal was completely replaced in the diet with BSF meal. In the latter study, the yolk content in α-tocopherol increased by 4.21%, but in this case, the difference between the soybean and the BSF diet did not reach statistical significance. The increase in the yolk tocopherols content resulting from the replacement of soybean meal with BSF meal in the diet of laying hens should have been expected since the concentration of tocopherols in BSF larvae is much higher than in soybean meal [41,73]. Although in the study of Secci et al. [25] yolk retinol concentration was not influenced by the dietary replacement of soybean meal with BSF meal, the decrease in yolk retinol concentration noticed in the present work can certainly be associated with the very low retinol content in BSF larvae [74].

Both lutein and zeaxanthin content in egg yolk increased linearly as dietary dose of BSF meal rose. Likewise, Secci et al. [25] found that yolk content in lutein was increased when soybean meal was replaced with BSF meal in the diet of laying hens. This modification of diet composition also resulted in an 11% higher yolk concentration of zeaxanthin in the latter publication, but for this xanthophyll, the difference did not achieve statistical significance. Since hens cannot synthesise xanthophylls, the lutein and zeaxanthin present in the egg yolk originate from the feed consumed by hens. Both in the present study and in that of Secci et al. [25], the inclusion of BSF meal in the diet resulted in a higher dietary content of lutein and zeaxanthin. On the one hand, this could explain why lutein and zeaxanthin yolk concentrations were higher in the eggs laid by hens that had consumed diets containing BSF meal. Furthermore, it has been observed that α-tocopherol improves carotenoid absorption since the dietary supplementation with α-tocopherol in laying hens resulted in greater accumulation of both lutein and zeaxanthin in egg yolk [57,75]. In the current work, the full replacement of soybean meal with BSF meal in the diet increased the yolk content in α-tocopherol. The latter could also have contributed to the enhancement of the amount of lutein in the egg yolk. Actually, it can be seen that a parallelism existed in the present study between yolk content in α-tocopherol and that of lutein and zeaxanthin. Obtaining lutein- and zeaxanthin-enriched eggs is all the more interesting as these eggs could be deemed a functional food since lutein and zeaxanthin have been associated with the prevention of cataracts and age-related macular degeneration [76,77].

Feeding laying hens with diets including BSF meal did not alter the yolk content in iron and phosphorus, but the yolk concentration in zinc linearly decreased with increasing dietary dose of BSF meal. In a previous study [45], it was also found that yolk iron concentration was unaffected by the dietary inclusion of BSF meal (with dietary doses of BSF meal up to 210 g/kg), but as regards zinc, its concentration in yolk showed no significant differences among diets, while its concentration in egg white was linearly reduced with increasing dietary content in BSF meal. The wide range of values reported for the zinc content in BSF (56–120 mg/kg) [45,74] could account for the different effects observed on yolk zinc concentration when feeding BSF meal to laying hens. Nevertheless, the decreasing zinc concentrations either in yolk or in albumen due to the dietary replacement of soybean meal with BSF meal are difficult to explain because the zinc content in BSF meal is higher than in soybean meal. Hence, further research is needed to elucidate the reasons for this reduction of zinc content in eggs.

It is commonly admitted that the fat profile in egg yolk depends to a great extent on the fat composition of the diet laying hens are fed. That’s why egg yolks can be enriched with ω-3 fatty acids by feeding laying hens with a diet including a source of these fatty acids, such as linseed [7]. In the current study, the dietary inclusion of BSF meal increased the proportion of monounsaturated fatty acids both in the diet and in the egg yolk, mostly due to the increase in the proportions of oleic acid and palmitoleic acid. In parallel, including BSF meal in the diet reduced the proportion of polyunsaturated fatty acids in the diet and in the egg yolk as well. The latter was mainly due to a reduction in the proportion of ω-6 fatty acids, specifically a decrease in the proportion of linoleic acid. Very few studies have evaluated so far the effect of feeding BSF meal on the egg yolk fatty acid profile. Besides, taking into account the variability that exists as regards fat profile in BSF resulting from the different substrates that can be used to raise BSF, the comparison with the results of other research works turns out to be difficult. For instance, the BSF larvae used in the study of Lokaewmanee et al. [61] were richer in linoleic acid than the BSF meal used in the present trial, and this difference in BSF fatty acid profile entailed different results between studies for the egg yolk fatty acid profile. While in both studies feeding the laying hens with BSF reduced the yolk proportion of γ-linolenic acid (C18:3 ω-6) and that of docosahexaenoic acid (C22:6 ω-3), the reduction in the yolk proportion of ω-6 fatty acids did not reach statistical significance in the work of Lokaewmanee et al. [61].

## 5. Conclusions

Soybean meal could be fully replaced by black soldier fly meal in the diet of free-range reared laying hens without affecting the laying rate, the feed intake or the feed conversion ratio of hens. The partial or full replacement of the main dietary protein source neither influenced egg weight, eggshell thickness or the yolk contents in fat, protein, cholesterol, choline, B vitamins, cholecalciferol, iron and phosphorus. The yolk concentrations in α- and γ-tocopherol and in lutein and zeaxanthin were greater in eggs from hens fed diets containing black soldier fly meal. Nevertheless, the dietary inclusion of black soldier fly meal entailed some drawbacks like a reduction of the yolk colour score and the albumen Haugh units, decreased retinol content in egg yolk, and a negative linear effect on egg yolk zinc concentration and on the proportions of polyunsaturated and ω-3 fatty acids with increasing dietary dose of black soldier fly meal. Since in the Mediterranean countries, consumers attach great importance to yolk colour; further research should be conducted to elucidate how the decreasing effect on yolk colour score due to the dietary inclusion of black soldier fly meal could be balanced out.

## Figures and Tables

**Table 1:** Analysed nutrient composition of soybean and black soldier fly meals (g/kg as is, if not indicated otherwise).

Nutrient	Soybean Meal	Black Soldier Fly Meal
Crude protein	421	559
Fat	13.1	70.0
Crude fibre	39.0	103
Calcium	3.80	26.5
Phosphorus	7.70	13.4
Sodium	0.10	1.30
Total carotenoids (mg/kg)	1.80	5.30
Lutein (mg/kg)	1.16	1.01
Zeaxanthin (mg/kg)	0.089	1.21
β-carotene (mg/kg)	0.374	0.867
α-,β-cryptoxanthin (mg/kg)	ND ^1^	0.597
Amino acids		
Alanine	19.8	34.3
Arginine	32.1	28.8
Aspartic acid	51.7	55.5
Cysteine	3.60	7.00
Glutamic acid	80.2	65.9
Glycine	18.2	31.2
Histidine	10.7	17.9
Isoleucine	14.5	18.0
Leucine	34.0	39.3
Lysine	27.2	33.7
Methionine	5.10	7.00
Phenylalanine	21.6	23.8
Proline	23.2	31.4
Serine	23.2	23.8
Threonine	17.6	21.6
Tryptophan	6.00	9.40
Tyrosine	19.7	33.0
Valine	19.8	32.6
Fatty acid profile (%)		
Lauric acid (C12:0)	ND	30.9
Myristic acid (C14:0)	0.20	6.73
Palmitic acid (C16:0)	13.6	17.5
Stearic acid (C18:0)	4.08	5.00
Oleic acid (C18:1 ω-9)	9.38	18.7
Linoleic acid (C18:2 ω-6)	55.2	13.7
Linolenic acid (C18:3 ω-3)	3.78	0.98
Saturated fatty acids	18.3	61.4
Monounsaturated fatty acids	20.7	23.4
Polyunsaturated fatty acids	61.0	15.2
ω-6 fatty acids	57.2	13.7
ω-3 fatty acids	3.78	1.48

^1^ ND = Not Detected.

**Table 2:** Ingredient and nutrient compositions of the experimental diets (g/kg as fed, if not indicated otherwise).

Ingredients	Experimental Diets		
	Control	80 g/kg BSF meal	160 g/kg BSF meal
Barley	316	330	356
Wheat	200	200	200
Soybean meal	210	105	0.0
Black soldier fly (BSF) meal	0.0	80.0	160
Sunflower meal (30% crude protein)	100	100	100
Soybean oil	60.0	60.0	60.0
Calcium carbonate	96.3	104	102
Monocalcium phosphate	7.30	8.60	10.9
Salt	3.40	4.20	3.70
L-Lysine	0.40	0.90	0.80
DL-Methionine	1.60	2.30	1.60
Vitamin-mineral premix ^1^	5.00	5.00	5.00
Analysed composition			
Starch	284	297	316
Fat	73.2	77.7	82.4
Crude fibre	51.1	55.9	61.3
Crude protein	176	179	182
Lysine	8.90	9.10	8.90
Methionine	3.90	4.50	4.00
Ash	134	147	148
Calcium	40.0	45.1	46.1
Phosphorus	6.00	6.60	7.50
Sodium	1.50	1.90	1.80
Total carotenoids (mg/kg)	15.4	19.0	19.0
Lutein (mg/kg)	5.01	6.05	6.18
Zeaxanthin (mg/kg)	5.10	6.71	7.03
β-carotene (mg/kg)	1.74	1.86	1.55
α-,β-cryptoxanthin (mg/kg)	2.05	2.68	2.23
Fatty acid profile (%)			
Oleic acid (C18:1 ω-9)	30.1	47.0	45.4
Linoleic acid (C18:2 ω-6)	51.7	30.8	30.3
Linolenic acid (C18:3 ω-3)	2.18	2.90	2.86
Saturated fatty acids	14.2	17.2	19.1
Monounsaturated fatty acids	31.4	48.5	47.1
Polyunsaturated fatty acids	54.4	34.3	33.8
ω-6 fatty acids	52.2	31.3	30.7
ω-3 fatty acids	2.18	3.03	3.08
Calculated composition			
AME ^2^ (MJ/kg)	11.3	11.3	11.6

^1^ Vitamin-mineral mix supplied the following per kilogram of diet: sulphur, 0.15 g; vitamin A, 10,000 IU; vitamin D_3_, 2000 IU; vitamin E, 20.0 IU; vitamin K, 3.00 mg; thiamine, 1.00 mg; riboflavin, 5.00 mg; vitamin B6, 2.00 mg; vitamin B_12_, 30.0 μg; niacinamide, 30.0 mg; pantothenic acid, 6.44 mg; folic acid, 1.00 mg; biotin, 100 μg; choline, 150 mg; Fe, as FeSO_4_, 25.0 mg; Zn, as ZnO, 60.0 mg; Mn, as MnO, 100 mg; Cu, as CuSO_4_, 4.00 mg; I, as KI, 1.50 mg; Se, as Na_2_SeO_3_, 0.20 mg: phytase, 900 FTU; β-glucanase, 152 U; and β-xylanase, 1220 U; ^2^ AME = apparent metabolisable energy.

**Table 3:** Effect of dietary inclusion of black soldier fly (BSF) meal on egg-laying performance in 36- to 44-week-old laying hens.

	Experimental Diets			SEM ^1^	*p*-Value	Linear Effect	Quadratic Effect
	Control	80 g/kg BSF meal	160 g/kg BSF meal				
Daily egg production (%)	94.4	93.9	91.9	1.08	0.23	0.10	0.57
Average egg weight (g)	62.5	60.9	61.9	0.644	0.52	0.39	0.84
Daily egg mass (g/d)	58.9	57.1	56.9	0.777	0.13	0.10	0.43
Feed intake (g/d)	126.1	126.3	126.4	0.336	0.89	0.62	0.97
Feed conversion ratio (g feed/g egg mass)	2.15	2.22	2.24	0.074	0.68	0.40	0.78

^1^ n = 6 replicates per treatment (7 hens per replicate).

**Table 4:** Effect of dietary inclusion of black soldier fly (BSF) meal on egg weight, thickness and proportion of eggshell, yolk colour score and albumen Haugh units.

	Experimental Diets			SEM ^1^	*p*-Value	Linear Effect	Quadratic Effect
	Control	80 g/kg BSF meal	160 g/kg BSF meal				
Egg weight (g)	62.4	60.8	62.0	0.784	0.33	0.68	0.15
Shell thickness (μm)	357	360	358	5.42	0.92	0.81	0.75
Shell (%)	11.6	11.7	11.6	0.151	0.83	0.99	0.54
Yolk colour score	8.19 ^a^	3.89 ^b^	4.11 ^b^	0.198	<0.001	<0.001	<0.001
Haugh units at 0 days of storage ^2^	96.7 ^a^	84.4 ^b^	85.7 ^b^	1.64	<0.001	<0.001	0.0011
Haugh units at 14 days of storage	83.6	77.6	82.1	2.40	0.20	0.66	0.082
Haugh units at 30 days of storage	83.6	79.7	81.5	1.64	0.25	0.38	0.16

^a,b^ Means within a row with different superscripts differ significantly (*p* < 0.05); ^1^ n = 36 eggs per treatment; ^2^ Eggs were stored in darkness at 4 °C.

**Table 5:** Effect of dietary inclusion of black soldier fly (BSF) meal on yolk content in fat, crude protein, cholesterol, choline, vitamins, carotenoids and minerals.

	Experimental Diets			SEM ^1^	*p*-Value	Linear Effect	Quadratic Effect
	Control	80 g/kg BSF meal	160 g/kg BSF meal				
Fat (% DM)	62.6	63.2	62.8	0.378	0.56	0.79	0.31
Crude protein (% DM)	31.5	32.0	32.1	0.205	0.12	0.17	0.37
Cholesterol (mg/100 g DM)	1995	1982	1987	10.3	0.70	0.61	0.51
Choline (mg/100 g DM)	590	587	600	20.4	0.89	0.72	0.75
Vitamins							
Biotin (μg/g DM)	1.23	1.57	1.37	0.120	0.22	0.52	0.12
Folate (μg/100 g DM)	27.9	28.3	28.6	0.522	0.67	0.36	0.88
Cobalamin (μg/100 g DM)	5.68	5.64	5.49	0.184	0.74	0.44	0.82
Retinol (μg/g DM)	10.8 ^a^	9.82 ^b^	9.60 ^b^	0.234	0.011	0.045	0.21
Cholecalciferol (μg/g DM)	7.72	7.05	7.05	0.425	0.46	0.27	0.53
α-tocopherol (μg/g DM)	116 ^b^	107 ^b^	148 ^a^	7.51	0.0086	0.11	0.023
γ-tocopherol (μg/g DM)	7.75 ^b^	9.75 ^a^	9.75 ^a^	0.479	0.024	0.022	0.12
Total carotenoids (mg/kg)	16.0 ^b^	20.5 ^ab^	25.7 ^a^	1.68	0.018	0.0031	0.86
Lutein (mg/kg)	9.84 ^b^	10.8 ^ab^	12.1 ^a^	0.484	0.042	0.0091	0.77
Zeaxanthin (mg/kg)	5.97 ^b^	8.77 ^a^	10.6 ^a^	0.640	0.0061	0.0010	0.57
α-,β-cryptoxanthin (mg/kg)	ND ^2^	1.39	1.83	0.158	0.12	--	--
Minerals							
Iron (mg/kg DM)	110	111	112	2.39	0.76	0.45	0.93
Zinc (mg/kg DM)	79.0 ^a^	73.2 ^b^	70.7 ^c^	0.773	<0.001	<0.001	0.12
Phosphorus (% DM)	1.06	1.10	1.10	0.030	0.56	0.36	0.56

^a–c^ Means within a row with different superscripts differ significantly (*p* < 0.05); ^1^ n = 9 pools of 4 yolks/pool per treatment; ^2^ ND = Not Detected.

**Table 6:** Effect of dietary inclusion of black soldier fly (BSF) meal on the egg yolk fatty acid profile (% of total fatty acids).

	Experimental Diets			SEM ^1^	*p*-Value	Linear Effect	Quadratic Effect
	Control	80 g/kg BSF meal	160 g/kg BSF meal				
C14:0	0.267 ^c^	0.610 ^b^	0.750 ^a^	0.0059	<0.001	<0.001	<0.001
C14:1	ND ^2^	0.127 ^b^	0.198 ^a^	0.0038	<0.001	<0.001	-
C15:0	ND	0.087	0.082	0.0048	0.49	0.49	-
C16:0	23.4 ^b^	24.2 ^a^	24.6 ^a^	0.207	0.023	0.22	0.70
C16:1	2.81 ^b^	3.91 ^a^	4.26 ^a^	0.123	<0.001	0.12	0.36
C17:0	0.340 ^a^	0.220 ^b^	0.237 ^b^	0.010	<0.001	0.49	0.19
C17:1	0.122 ^a^	0.110 ^b^	0.127 ^a^	0.0031	0.0089	0.48	0.16
C18:0	8.55 ^a^	6.97 ^b^	7.09 ^b^	0.062	<0.001	0.55	0.30
C18:1	37.8 ^c^	42.6 ^b^	43.7 ^a^	0.252	<0.001	<0.001	<0.001
C18:2 ω-6	21.9 ^a^	16.8 ^b^	15.0 ^c^	0.444	<0.001	<0.001	0.011
C18:3 ω-3	0.607 ^b^	0.680 ^a^	0.610 ^b^	0.015	0.0013	0.27	0.61
C18:3 ω-6	0.198 ^a^	0.117 ^b^	0.102 ^c^	0.0042	<0.001	<0.001	<0.001
C20:1	0.207 ^b^	0.260 ^a^	0.263 ^a^	0.0051	<0.001	0.079	0.35
C20:2 ω-6	0.230 ^a^	0.210 ^a^	0.160 ^b^	0.011	0.0049	0.87	0.31
C20:3 ω-3	2.17 ^a^	1.76 ^b^	1.61 ^c^	0.037	<0.001	<0.001	0.019
C22:6 ω-3	0.792 ^a^	0.772 ^a^	0.627 ^b^	0.017	<0.001	0.37	0.17
C24:0	0.220 ^a^	0.177 ^b^	0.182 ^b^	0.0039	<0.001	0.76	0.49
C24:1	0.387	0.390	0.402	0.011	0.59	0.93	0.71
Saturated fatty acids (%)	32.8	32.3	33.0	0.192	0.089	0.51	0.23
Monounsaturated fatty acids (%)	41.3 ^c^	47.4 ^b^	48.9 ^a^	0.363	<0.001	<0.001	<0.001
Polyunsaturated fatty acids (%)	25.9 ^a^	20.3 ^b^	18.1 ^c^	0.513	<0.001	<0.001	0.023
ω-6 fatty acids (%)	22.3 ^a^	17.1 ^b^	15.3 ^c^	0.458	<0.001	<0.001	0.013
ω-3 fatty acids (%)	3.57 ^a^	3.21 ^b^	2.80 ^c^	0.062	<0.001	<0.001	0.73
Ratio ω-6/ω-3	6.26 ^a^	5.31 ^b^	5.47 ^b^	0.070	<0.001	0.31	0.66

^a–c^ Means within a row with different superscripts differ significantly (*p* < 0.05); ^1^ n = 9 pools of 4 yolks/pool per treatment; ^2^ ND = Not Detected.

## Data Availability

The data presented in this study are available on request from the corresponding author.
